# Emergence of carbapenem-resistant ST131 *Escherichia coli* carrying *bla*_OXA-244_ in Germany, 2019 to 2020

**DOI:** 10.2807/1560-7917.ES.2020.25.46.2001815

**Published:** 2020-11-19

**Authors:** Sybille Welker, Sébastien Boutin, Thomas Miethke, Klaus Heeg, Dennis Nurjadi

**Affiliations:** 1 Institute for Medical Microbiology and Hygiene, Medical Faculty Mannheim of Heidelberg University, Mannheim, Germany; 2 Medical Microbiology and Hygiene, Department of Infectious Diseases, Heidelberg University Hospital, Heidelberg, Germany

**Keywords:** Escherichia coli ST131, OXA-244, carbapenem resistance, Enterobacterales, antimicrobial resistance

## Abstract

The dissemination of carbapenem-producing Gram-negative bacteria is a major public health concern. We report the first detection of OXA-244-producing ST131 O16:H5 *Escherichia coli* in three patients from two tertiary hospitals in the south-west of Germany. OXA-244 is emerging in Europe. Because of detection challenges, OXA-244-producing *E. coli* may be under-reported. The emergence of carbapenem resistance in a globally circulating high-risk clone, such as ST131 *E. coli* is of clinical relevance and should be monitored closely.

*Escherichia coli* of the ST131 lineage is considered as a successful and emerging high-risk pandemic multidrug-resistant *E. coli* strain [1,2]. Typically, most ST131 *E. coli* are resistant to third-generation cephalosporins but remain susceptible to carbapenems [1]. We detected three OXA-244-producing ST131 *E. coli* from patient samples in two tertiary hospitals in the south-west of Germany between January 2019 and June 2020. 

OXA-244 is a single-point mutation variant (Arg214Gly) of the globally circulating OXA-48 [3], resulting in lower minimum inhibitory concentration (MIC) values, which poses a major challenge for its detection [4,5]. 

The aim of our study was to investigate the genetic diversity of the emerging OXA-244-producing *E. coli* in the Rhine-Neckar region using whole-genome sequencing. 

## Local surveillance measures for multidrug-resistant organisms

Since January 2019, the University Hospitals in Heidelberg and Mannheim, located in the south-west of Germany (Rhine-Neckar region), have implemented routine molecular typing by whole-genome sequencing (WGS) of non-repetitive multidrug-resistant Gram-negative bacteria (MDR-GN) from admission screening and clinical samples as part of the local infection control measures. Admission rectal screening for MDR-GN was performed for all risk patients, which includes (i) admission to intermediate and intensive care units, (ii) previous colonisation with multidrug-resistant organisms (MDRO) or contact with MDRO patients, (iii) contact with a high-prevalence setting or endemic region for MDRO (including travel and migration), (iv) chronic wounds and (v) close contact to animals, as previously described [6]. The cultural detection methods used a selective medium (ChromID ESBL, Biomérieux, Nürtingen, Germany) and were confirmed by antibiotic susceptibility testing (AST) with VITEK2 (Biomérieux) interpreted according to the European Committee on Antimicrobial Susceptibility Testing (EUCAST) clinical breakpoints v10.0 [7]. Carbapenemase genes were detected with an in-house PCR (data not shown) of all isolates with phenotypic resistance to carbapenem or with suspected carbapenem resistance (i.e. elevated MIC for carbapenems).

Only the first detected isolate from each patient was sequenced. Molecular characterisation was performed by short-read WGS using the Nextera DNA Flex Library Prep Kit (Illumina, San Diego, United States) and the MIseq instrument (2 × 300 bp), as described previously [6]. Assembly was performed with Spades 3.13.0 [8]. Core genome was calculated using Roary [9] after annotation with Prokka 1.14.1 [10]. Coverage for each contig was extracted from the Spades output. Resistance genes were annotated using Abricate 1.0.0 with the database form the National Center for Biotechnology Information (NCBI) [11], the comprehensive antibiotic resistance database CARD [12], Antibiotic resistance gene-ANNOTation (ARG-ANNNOT) [13] and Resfinder 3.0 [14] (latest update on 10 June 2020). Subtyping of the serotype and *fimH* was performed using SeroTypeFinder 2.0 (https://cge.cbs.dtu.dk/services/SerotypeFinder/) and FimTyper 1.0 (https://cge.cbs.dtu.dk/services/FimTyper/). Assembled draft genome sequences are deposited in the NCBI GenBank database under the bioproject number PRJNA546126.

## Molecular and microbiological characteristics of OXA-244-producing *Escherichia coli*

Between January 2019 and June 2020, we identified 50 *E. coli* with phenotypic carbapenem resistance, of which 41 carried a carbapenemase. Nine of the 41 carried *bla*_OXA-244_, which belonged to three clonal lineages ST38 (n = 5), ST131 (n = 3) and ST167 (n = 1). The isolate belonging to ST167 haboured two carbapenemase genes, *bla*_NDM-5_ and *bla*_OXA-244_. Relevant clinical and microbiological characteristics of the nine patients are summarised in Table 1.

**Table 1:** Patient, clinical and microbiological characteristics of blaOXA-244 harbouring *Escherichia coli* in Heidelberg and Mannheim, Germany, 2019–2020 (n = 9)

Patient number	Patient characteristics						Clinical and microbiological characteristics							
	Accession number^a^	First detection	Detection on admission	Age group (years)	Migration /travel	Specimen^b^	Colonisation	Infection	Serotype^c^	MLST^d^	Carbapenemase	*fimH* ^e^	Meropenem MIC (µg/mL)^f^	CIM
P1	SAMN16521172	Oct 2019	Yes	<10	No	Rectal swab	+	−	O16:H5	ST131	OXA-244	41	0.125^g^	+
P2	SAMN16521173	Jan 2020	No	<10	No	Rectal swab, urine	+	+	O16:H5	ST131	OXA-244	41	0.75^g^	+
P3	SAMN16521174	Dec 2019	No	≥70	Libya^g^	Rectal swab, urine, blood culture	+	+	O16:H5	ST131	OXA-244	41	6^g^	+
P4	SAMN16521175	Sep 2019	Yes	40–50	Unknown	Rectal swab	+	−	O86:H14	ST38	OXA-244	−	0.5	+
P5	SAMN16521176	Dec 2019	Yes	40–50	No	Rectal swab	+	−	O86:H14	ST38	OXA-244	−	0.19	+
P6	SAMN16521177	Mar 2020	Yes	20–30	Unknown	Rectal swab	+	−	O102:H6	ST38	OXA-244	5	0.75	+
P7	SAMN16521178	May 2020	No	<10	No	Rectal swab	+	+	O153:H30	ST38	OXA-244	5	0.38	+
P8	SAMN16521179	Dec 2019	No	≥70	No	Rectal swab, urine	+	+	O86:H18	ST38	OXA-244	−	0.5	+
P9	SAMN16521180	Jul 2020	Yes	60–70	Unknown	Rectal swab	+	−	O101:H17	ST167	OXA-244 + NDM-5	−	4^h^	+

CIM: carbapenem inactivation assay; MIC: minimum inhibitory concentration; MLST: multilocus sequence type; +: positive; −: negative.

^a^ Sequences were deposited in the Genbank at NCBI under the Bioproject PRJNA546126.

^b^ First detection specimen is underlined, in cases with multiple samples. Only the first isolate of each patient was sequenced.

^c^ Serotype was derived from the assembled draft genome using the CGE Serotype Finder 2.0 (https://cge.cbs.dtu.dk/services/SerotypeFinder/).

^d^ MLST derived from the assembled draft genome using CGE FimTyper 1.0 (https://cge.cbs.dtu.dk/services/FimTyper/).

^e^ *fimH* typing was derived from the assembled draft genome using the CGE Serotype Finder V2.0.

^f^ Meropenem MIC was determined using an agar-based gradient diffusion test (E-test).

^g^ E-test exhibited slight growth within the zone of inhibition.

^h^ Patient was in Libya prior to detection of OXA-244 producing *E. coli*.

**Figure 1:**
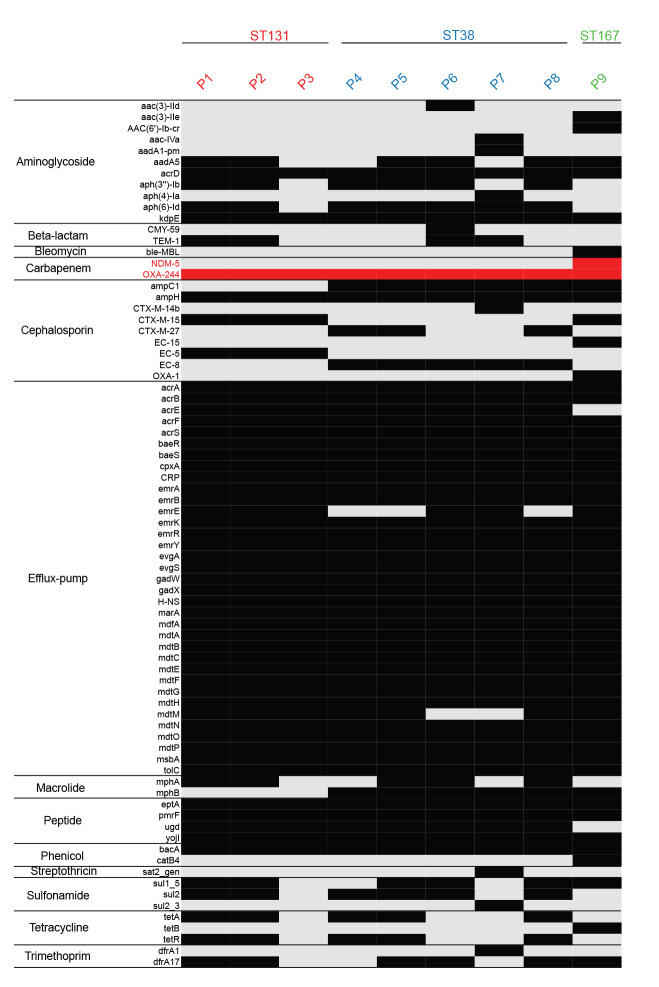
Antimicrobial resistance genes in OXA-244-producing *Escherichia coli* in the Rhine-Neckar region, Germany, 2019–2020 (n = 9)

The presence of genotypic antibiotic resistance determinants is summarised in Figure 1. Antibiotic susceptibility of all *bla*_OXA-244_ is displayed in Table 2. Isolates of the ST38 lineage carried variable extended-spectrum β-lactamase (ESBL) genes, such as *bla*_CTX-M-14_, *bla*_CTX-M-27_ and *bla*_TEM-1_, whereas all isolates of the ST131 clonal lineage harboured *bla*_CTX-M-15_ in addition to the *bla*_OXA-244_ gene (Figure 1). 

ST: sequence type.

Black squares: presence, grey squares: absence of antimicrobial resistance genes; red font and red squares: carbapenemase genes.

**Table 2:** Antibiotic susceptibility profile of *bla*_OXA-244_-harbouring *Escherichia coli* in the Rhine-Neckar region, Germany, 2019–2020 (n = 9)

Substance	ST131						ST38										ST167	
	P1		P2		P3		P4		P5		P6		P7		P8		P9	
	MIC	Int	MIC	Int	MIC	Int	MIC	Int	MIC	Int	MIC	Int	MIC	Int	MIC	Int	MIC	Int
Piperacillin/tazobactam	≥ 128	R	≥ 128	R	≥ 128	R	≥ 128	R	≥ 128	R	≥ 128	R	≥ 128	R	≥ 128	R	≥ 128	R
Cefotaxim	≥ 64	R	≥ 64	R	≥ 64	R	≥ 64	R	≥ 64	R	8	R	≥ 64	R	≥ 64	R	≥ 64	R
Ceftazidim	32	R	32	R	≥ 64	R	8	R	8	R	8	R	8	R	16	R	≥ 64	R
Cefepim	16	R	16	R	≥ 32	R	16	R	8	R	≤ 0.12	S	≥ 32	R	4	I	≥ 32	R
Ceftolozan/tazobactam	4	R	4	R	≥ 32	R	1	S	1	S	4	R	8	R	1	S	≥ 32	R
Imipenem	≤ 0.25	S	1	S	2	S	≤ 0.25	S	0.5	S	≤ 0.25	S	0.5	S	0.5	S	≥ 16	R
Meropenem	≤ 0.25	S	≤ 0.25	S	≥ 16	R	≤ 0.25	S	≤ 0.25	S	≤ 0.25	S	1	S	0.5	S	8	I
Ciprofloxacin	≤ 0.25	S	≤ 0.25	S	2	R	≤ 0.25	S	≤ 0.25	S	≤ 0.25	S	1	R	≤ 0.25	S	≥ 4	R
Trimethoprim/sulfamethoxazole	≥ 320	R	≥ 320	R	≤ 20	S	≤ 20	S	≥ 320	R	≥ 320	R	≥ 320	R	≥ 320	R	≥ 320	R
Gentamicin	≤ 1	S	≤ 1	S	≤ 1	S	≤ 1	S	≤ 1	S	≥ 16	R	≥ 16	R	≤ 1	S	≥ 16	R
Tobramycin	≤ 1	S	≤ 1	S	≤ 1	S	≤ 1	S	≤ 1	S	4	R	≥ 16	R	≤ 1	S	8	R
Amikacin	2	S	2	S	2	S	2	S	2	S	2	S	2	S	≤ 1	S	2	S
Tigecyclin	≤ 0.5	S	≤ 0.5	S	≤ 0.5	S	≤ 0.5	S	≤ 0.5	S	≤ 0.5	S	≤ 0.5	S	≤ 0.5	S	≤ 0.5	S
Aztreonam	≥ 64	R	≥ 64	R	≥ 64	R	16	R	16	R	2	I	16	R	16	R	≥ 64	R
Fosfomycin	≤ 16	S	≤ 16	S	≤ 16	S	≤ 16	S	≤ 16	S	≤ 16	S	≤ 16	S	≤ 16	S	≤ 16	S
Colistin^a^	≤ 0.5	S	≤ 0.5	S	≤ 0.5	S	≤ 0.5	S	≤ 0.5	S	≤ 0.5	S	≤ 0.5	S	≤ 0.5	S	≤ 0.5	S

I: intermediate; Int: interpretation; MIC: minimum inhibitory concentration in mg/L; R: resistant; S: susceptible; ST: sequence type.

MIC was determined by VITEK2 using the AST-N389 test panel. Antibiotic susceptibility was interpreted using the EUCAST clinical breakpoints v10.0 [7].

^a^ Interpretation and MIC for colistin using VITEK2 may not be reliable [28].

**Figure 2:**
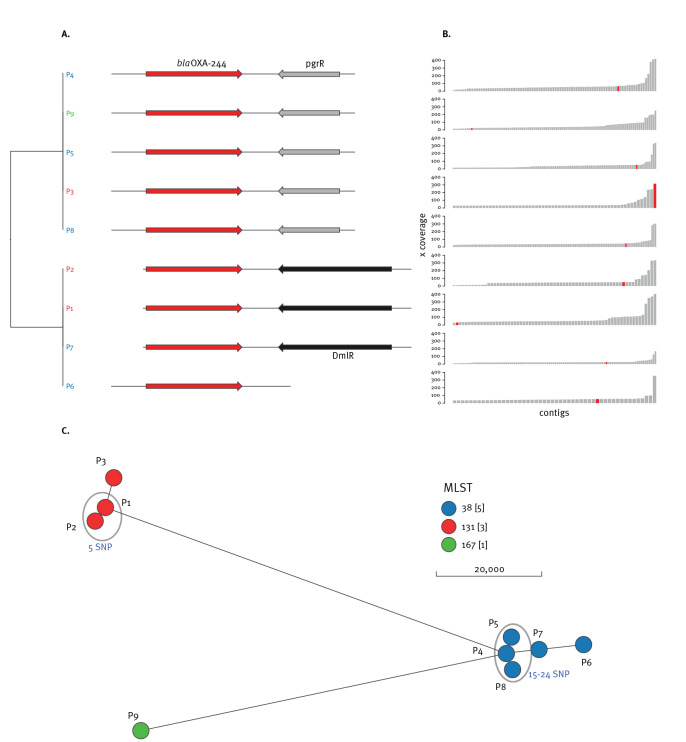
Genetic characteristics of *bla*_OXA-244_-harbouring *Escherichia coli* in the Rhine-Neckar region, Germany, 2019–2020 (n = 9)

MLST: multilocus sequence type; SNP: single-nucleotide polymorphism.

Consistent with published data, the *bla*_OXA-244_ genes are most likely to have been integrated into the chromosome because sequencing coverage of the blaOXA-244-containing contigs was lower than the overall average sequencing coverage (Figure 2A and 2B) [5,15]. 

A. Clustering by the genetic environment of the *bla*_OXA-244_ gene revealed two clusters. We were not able to identify any transposable elements within the contig of the assembled draft genome. Isolates belonging to the ST131 *E. coli* are indicated in red, ST38 in blue and ST167 in green font, corresponding to the figure legend of the minimum-spanning tree in panel C.

B. The analysis of the coverage in comparison to other contigs in the assembled draft genome suggests a chromosomal integration of the *bla*_OXA-244_ gene in almost all isolates. Red vertical bars represent the *bla*_OXA-244_-containing contig.

C. Minimum-spanning tree based on the core genome of all sequenced *E. coli* in this study. Potential transmission clusters are indicated by the grey circles with SNP differences over the core genome in blue. There was no indication of patient-to-patient transmission (3,413 genes, 108,017 polymorphic sites). Numbers in square brackets indicate the number of isolates belonging to the MLST.

Seven of nine isolates were susceptible to meropenem as indicated by the low MIC in two different AST methods (Tables 1 and 2). One isolate (ST167, P9) carried both *bla*_OXA-244_ and *bla*_NDM-5_ so that high MIC values for carbapenem were expected. However, the isolate from P3 exhibited an unusually high MIC for meropenem for an OXA-244 producer in both AST methods (≥ 16 mg/L in VITEK and 6 mg/L in E-test) (Tables 1 and 2), for reasons we could not explain. Nevertheless, all nine isolates exhibited positive results in the phenotypic carbapenem inactivation assay (CIM) using meropenem disk (10 µg) with a 2 h inactivation step [16]. Our findings suggest that CIM may be a reliable method to detect OXA-244 producers and should be validated in further studies.

## Potential origin and nosocomial transmission of OXA-244-producing ST131 *Escherichia coli*

SNP analysis to evaluate the clonal relationship of the isolates suggested two potential transmission clusters of patients P1-P2 with five SNP and P4-P5-P8 with 15–24 SNP (Figure 2C). Patient P1 was colonised with *bla*_OXA-244_ *E. coli* on admission. There was no recent travel exposure so that community acquisition in Germany was possible. P2 stayed in the same ward as P1 with some temporal overlap. P2 was born in the hospital and acquired the colonisation with ST131 OXA-244-producing *E. coli* during the hospital stay. Nosocomial transmission is a very likely source of acquisition as suggested by the identical genotypic and phenotypic resistance of both isolates of P1 and P2 (Figure 1 and Table 2). P3 was in a different hospital than P1 and P2. The lack of epidemiological link is consistent with the genomic analysis, which did not indicate transmission. P3 had had contact with the healthcare system in Libya and was initially screened negative on admission in Germany. The *bla*_OXA-244_ *E. coli* was detected in subsequent screenings. However, we cannot fully rule out importation because the sensitivity of the detection method is limited [15].

In the ST38 cluster, there was no epidemiological overlap so that a nosocomial patient-to-patient transmission event is unlikely. Nevertheless, community transmissions caused by clonal dissemination of *bla*_OXA-244_-positive ST38 *E. coli* in Germany cannot be entirely ruled out [17].

## Discussion

The increased incidence in Europe of community-acquired infections with *E. coli* carrying OXA-244 is of public health relevance as reflected by the rapid risk assessment by the European Centre for Disease Prevention and Control (ECDC) at the beginning of 2020 [18]. Recently, several federal states in Germany reported a rise in detection of community-acquired infections with ST38 OXA-244-producing *E. coli* [17]. Similar observations have been reported in other European countries [4,5,19-21]. 

In Germany and other neighbouring countries in Europe, *bla*_OXA-244_ is predominantly found in ST38 *E. coli* [4,17,19,21,22]. Surveillance data from Denmark and France reported the presence of *bla*_OXA-244_ in other clonal groups (ST10, ST38, ST69, ST167, ST10, ST361 and ST 3268) [21,23], but to the best of our knowledge the presence of *bla*_OXA-244_ in ST131 *E. coli* in Europe has not been reported before. Besides being responsible for serious extra-intestinal infections, the development of resistance to carbapenems in the ST131 *E. coli* clonal lineage, is particularly worrisome as carbapenems are often the last line of therapy for life-threatening infections [2,24]. There are no systematic data on the prevalence of carbapenemase-producing Gram-negative bacteria in the Rhine-Neckar region. However, our data suggest a low prevalence of 0.5% (131/27,387 screened patients in the Heidelberg University Hospital in 2019), which is consistent with published data [25].

Peirano et al. reported that the global incidence of carbapenemase-producing *E. coli* ST131 O25b:H4 of the *fimH*30/virotype C lineage is increasing, with *bla*_KPC_ as the most common carbapenem-resistance determinant [2]. In contrast, our *E. coli* ST131 has the serotype O16:H5 with *bla*_OXA-244_ that belongs to the *fimH*41/virotype C lineage [26]. Although the major lineage of the highly virulent ST131 belongs to the serotype O25b:H4 and *fimH*30, a murine infection model suggested that ST131 O16:H5 *fimH*41 is comparable to the H30 lineage in virulence and lethality [27], which implies that the emergence of carbapenems resistance in the H41 ST131 lineage is equally relevant. 

Our study has limitations, the detection of OXA-244 producing *E. coli* is a major diagnostic challenge owing to its low level of phenotypic resistance to carbapenems; therefore OXA-244 producers may be underreported. Nevertheless, our finding suggests that a simple phenotypic assay for carbapenem inactivation combined with routine WGS may be useful to detect low carbapenemase producers, such as OXA-244. In addition, the epidemiological data of our patients were limited so that the exact origin of the OXA-244-producing ST131 *E. coli* in this study cannot be fully elucidated.

## Conclusion

The emergence and dissemination of virulent and dominant *E. coli* clones with resistance to last-line antibiotics is a public health concern. Our findings emphasise the necessity of adequate surveillance measures and warrant further studies on the epidemiology and transmission dynamics of carbapenem-resistant *E. coli* both in the hospital and community setting.
